# Sweet spot for resting-state functional MRI effect of deep brain stimulation in dystonia lies in the lower pallidal area

**DOI:** 10.1016/j.nicl.2025.103750

**Published:** 2025-02-05

**Authors:** Pavel Filip, Andrej Lasica, Dimitra Kiakou, Karsten Mueller, Jiří Keller, Dušan Urgošík, Daniel Novák, Robert Jech

**Affiliations:** a Department of Neurology, First Faculty of Medicine, Charles University and General University Hospital in Prague, Kateřinská 30, 120 00 Prague, Czech Republic; b Center for Magnetic Resonance Research (CMRR), University of Minnesota, Minneapolis, MN, USA; c Department of Cybernetics, Czech Technical University in Prague, Prague, Czech Republic; d Max Planck Institute for Human Cognitive and Brain Sciences, Leipzig, Germany; e Department of Radiology, Na Homolce Hospital, Prague, Czech Republic; f Third Faculty of Medicine, Charles University in Prague, Prague, Czech Republic; g Department of stereotactic and radiation neurosurgery, Nemocnice Na Homolce, Prague, Czech Republic

**Keywords:** Deep brain stimulation, Dystonia, Internal globus pallidus, Resting-state functional magnetic resonance imaging

## Abstract

**Introduction:**

Deep brain stimulation (DBS) of the internal globus pallidus (GPi) is a well-established, effective treatment for dystonia. Substantial variability of therapeutic success has been the one of the drivers of an ongoing debate about proper stimulation site and settings, with several indications of the notional sweet spot pointing to the lower GPi or even subpallidal area.

**Methods:**

The presented patient-blinded, random-order study with cross-sectional verification against healthy controls enrolled 17 GPi DBS idiopathic, cervical or generalised dystonia patients to compare the effect of the stimulation in the upper and lower GPi area, with the focus on sensorimotor network connectivity and local activity measured using functional magnetic resonance.

**Results:**

Stimulation brought both these parameters to levels closer to the state detected in healthy controls. This effect was much more pronounced during the stimulation in the lower GPi area or beneath it than in slightly higher positions, with stimulation-related changes detected by both metrics of interest in the sensorimotor cortex, striatum, thalamus and cerebellum.

**Conclusions:**

All in all, this study not only replicated the results of previous studies on GPi DBS as a modality restoring sensorimotor network connectivity and local activity in dystonia towards the levels in healthy population, but also showed that lower GPi area or even subpallidal structures, be it white matter or even small, but essential nodes in the zona incerta as nucleus basalis of Meynert, are important regions to consider when programming DBS in dystonia patients.

## Highlights

• GPi DBS restores sensorimotor network connectivity and activity in dystonia.
• Sensorimotor cortex, striatum, thalamus and cerebellum are most prominent nodes.
• This effect is more pronounced when stimulating the lower GPi and subpallidal area.

## 1 Introduction

Deep brain stimulation (DBS) of the internal globus pallidus (GPi) is an effective and well-established therapeutic modality in movement disorders, especially dystonia (Volkmann et al., 2012, Vidailhet et al., 2005). However, the procedure is associated with non-responder rate of up to 25 % (Volkmann et al., 2012). This inopportune statistic is related partly to the complicated nature of the selection of eligible patients, partly to the titration of DBS parameters due to substantially delayed clinical response especially in tonic dystonic components, with a high risk of inappropriate stimulation settings and/or positions (Kupsch et al., 2011). Furthermore, there is a substantial variability in electrode placement, which may well be the most frequent driver for observed therapeutic failures (Pauls et al., 2017), combined with concerns about precise anatomical localisation to stimulate. This per se might be relatively surprising, since the neurophysiological basis is well researched and postulated as that of a network disorder encompassing cortico-basal ganglia-thalamo-cortical and cerebellar networks, although some debate still continues (Balint et al., 2018). Indeed, the precise position of the most effective stimulation point within the pallidal region, the coveted “sweet spot”, has been a matter of several studies. More posterior electrode placement (Tisch et al., 2007), the border between the posterior and middle third of GPi (Cheung et al., 2014) or even the area in the proximity of lamina interna above the ventral border of GPi (Schönecker et al., 2015) have been recommended, with tractography-based indications of divergence of optimal stimulation site in cervical and generalised dystonia patients (Horn et al., 2022). However, the most notable study based on over 100 GPi DBS dystonia patients points to the lower posterior GPi and subpallidal white matter tracts (Reich et al., 2019).

All these areas boast dense connections with regions highly relevant for dystonia pathophysiology, both premotor and motor cortex and major subcortical nodes of the classical model of basal ganglia circuitry (Fujita and Eidelberg, 2017). Although conventional clinical MRI scans usually fail to reveal any gross structural changes in these regions in dystonia patients, functional magnetic resonance imaging (fMRI) studies have repeatedly associated them with sensorimotor-processing and integration dysfunction hypothesised in dystonia (Simonyan, 2018), together with several spatially more distant structures essential for motor programming as cerebellum (Filip et al., 2013, Filip et al., 2017). fMRI also emerged as a viable tool able to detect the acute effects of GPi DBS in the sensorimotor network – increase in the connectivity of putamina, thalamus, cerebellum and several cortical areas towards the levels detected in healthy population (Filip et al., 2022) and also decrease in local activity in the sensorimotor cortex, combined with activity increase in pons and several prefrontal and parietal cortical areas (Loh et al., 2022).

The presented study aimed to build upon the previous work focusing on the fMRI effects of GPi DBS in idiopathic dystonia and to capitalise on this technique as a method to detect neural signature of the sweet spot hypothesised in the lower GPi area and subpallidal white matter. To ensure the continuity and potentially expand the previous findings in GPi DBS, two established resting-state fMRI (rs-fMRI) parameters were selected: as a connectivity measure, partial correlation eigenvector centrality (EC) – a metric supposedly superior to simpler connectivity measures with higher biological relevance (Lohmann et al., 2010); and as a local activity proxy, resting state physiological fluctuation amplitude (RSFA) – a metric similar to the previously published amplitude of low-frequency fluctuations, but with better documented biological pertinency (Kannurpatti and Biswal, 2008). Based on the hypothesised nature of clinical GPi DBS effect, only sensorimotor network was to be considered, which, especially for the connectivity analysis, was chosen to provide a more nuanced view on the pathophysiology and elicited DBS effects. The primary objectives were two-fold: A) to compare the state of the sensorimotor network between healthy controls and dystonia patients, thus establishing a baseline difference and nature of the pathological alteration to the considered rs-fMRI metrics; and B) to detect the eventual difference of DBS effect on these metrics when stimulating in the “lower GPi area” or slightly underneath i.e. the most ventral (lowest) DBS lead contact, which should correspond to the presumptive sweet spot location, and in the “upper GPi area”, i.e. utilising the contacts higher than the most ventral ones. We hypothesised that the stimulation in the lower GPi area would elicit stronger effect on the sensorimotor network than that in the upper GPi area and that the direction of the effect for both the rs-fMRI metrics would bring the sensorimotor network state closer to the condition detected in healthy controls.

## 2 Methods

### 2.1 Subjects

20 DY patients (10 females, median age [range] of 53 [24–75] years) managed with chronic GPi DBS therapy were enrolled into this study. The diagnosis of dystonia has been confirmed by a tertiary-care movement disorders centre specialist in accordance with the relevant diagnostic criteria (Albanese et al., 2013). Inclusion criteria were as follows: the presence and/or history of primary dystonia without head tremor (to avoid MRI artifacts), active bilateral GPi DBS as a part of dystonia management for at least 6 months before the participation in this study, stable DBS parameters for at least 1 month before the participation in this study. General exclusion criteria were as follows: neurological and/or psychiatric disorder other than dystonia, general contraindications to MRI examination, a non-negligible vascular or space occupying central nervous system lesion other than the implanted DBS system. Clinical data recorded within this study included the following: neurological status based on the referenced dystonia classification (axis I – clinical characteristics [age of onset, body distribution, temporal pattern and associated features] and axis II – aetiology [nervous system pathology, inherited/acquired status] and current medication (Albanese et al., 2013). The clinical condition was evaluated using the motor score subsections of either Burke-Fahn-Marsden Dystonia Scale (BFMDS) (Burke et al., 1985), or Toronto Western Spasmodic Torticollis Rating Scale (TWSTRS) (Consky et al., 1990) based on the clinical presentation (generalised or cervical dystonia, respectively), at four time points – before DBS implantation, before two DBS-ON rs-fMRI acquisitions and before DBS-OFF acquisition (see the Supplementary methods − Imaging Protocol). And lastly, information related to the DBS system was recorded (hardware information, time since the implantation, clinical response to DBS according to the attending neurologist and stimulation settings in the two DBS programs utilised in the study [stimulation mode (constant current or constant voltage), active contacts, amplitude, pulse width, frequency and therapy impedance]). The clinical effect of DBS was calculated as the ratio of the difference between the clinical scores (BFMDS or TWSTRS according to the clinical presentation) at relevant timepoints or DBS settings (positive values corresponded to clinical improvement and vice versa).

17 healthy controls (HC) (10 females, median age [range] of 58 [26–81]) were selected from the pool of previously acquired subjects (Mueller et al., 2017) utilising Matchit r-library based on age and sex of dystonia patients (Ho et al., 2011).

Every subject provided their written informed consent form in accordance with the Declaration of Helsinki. The study protocol was approved by the ethics committee of the General University Hospital in Prague.

### 2.2 Imaging protocol

The investigational MRI protocol acquired using a 1.5 Tesla Siemens Avanto System (Siemens, Erlangen, Germany) included a T1-weighted (T1w) structural scan and rs-fMRI session(s) (see Supplementary methods for details). While HC underwent only one rs-fMRI acquisition, DY patients underwent three rs-fMRI acquisitions in random order across the subjects, with patients blinded to the utilised DBS settings (the investigator was not blinded): A) DBS switched off (DBS-OFF session); B) active DBS utilising the stimulation parameters determined by the attending physician; C) active DBS with different contact settings to the previous condition, so that either the previous or this session used the lowest possible stimulation contacts. Pulse width and frequency were maintained, and the highest tolerable stimulation amplitude was set. Individual rs-fMRI acquisitions were separated by at least 20 min. No medication alterations or discontinuations were introduced as a part of the MRI protocol.

### 2.3 DBS electrode position analysis

The position of DBS electrode was analysed using Lead-DBS software (version 2.5.2) with the enhanced workflow (Horn and Kühn, 2015, Horn et al., 2019) (for further details, see Supplementary methods). Simple overlap of Volume of Tissue Activated (VTA) and the whole GPi and sensorimotor part of GPi (Ewert et al., 2018) was calculated.

### 2.4 Structural and functional MRI data analysis

Full MRI data processing pipeline is described in the Supplementary methods. Briefly, the Human Connectome Project (HCP) minimal preprocessing pipeline (Glasser et al., 2013) to derive warp to the common MNI space combined with the reconstructed brain surfaces into the standard Connectivity Informatics Technology Initiative (CIFTI) grayordinate space to be utilised for the subsequent rs-fMRI pipeline. rs-fMRI processing followed the HCP minimal preprocessing pipeline (Glasser et al., 2013) and the HCP rs-fMRI pipeline (Smith et al., 2013). Afterwards, processed rs-fMRI data were parcellated with a combination of HCP cortical parcellation (180 parcels per hemisphere) (Glasser et al., 2016), Oxford thalamic connectivity atlas (Behrens et al., 2003), Oxford-GSK-Imanova connectivity striatal atlas (Tziortzi et al., 2014) divided into the respective caudate and putamen subsegments based on FreeSurfer subcortical segmentation, probabilistic structural cerebellar atlas (Diedrichsen et al., 2009), and ATAG atlas (Keuken et al., 2017) for external and internal pallidum, red nucleus, substantia nigra and subthalamic nucleus. Only parcels of sensory-motor areas were considered in the further analysis (see Supplementary Fig. 1): somatosensory and motor cortex and premotor cortex (Glasser et al., 2016), motor-function-related cerebellar structures (lobules I-IV, V, VI, VIIIa, VIIIb, IX, crus I and II; dentate) (Guell and Schmahmann, 2020), sensory-motor parts of caudate and putamen, primary motor, premotor and sensory parts of thalamus, external and internal globus pallidus, red nucleus, substantia nigra and subthalamic nucleus; yielding 76 parcels altogether.

**Fig. 1:**
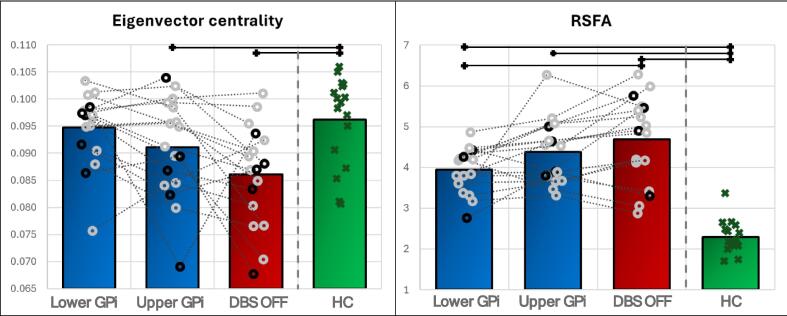
Comparison of DBS effect in the lower and upper GPi part (blue columns), DBS OFF state (red columns) in dystonia patients and healthy controls (green columns) for RSFA and eigenvector centrality for the whole sensorimotor network. Column heights correspond to group averages, values of individual dystonia patients are presented as grey and black (subjects with mirrored hemispheres) circles with jitter along the x-axis (to improve legibility and avoid overlaps), dotted lines connect the values of the three DBS states in each dystonia patient. Green crosses with jitter along the x-axis denote individual values of healthy controls. Statistically significant differences are marked with black horizontal lines above respective columns. Abbreviations: RSFA – resting state physiological fluctuation amplitude; HC – healthy controls; Lower and upper GPi – session with the stimulation of the lower and upper internal globus pallidus area, respectively; DBS-OFF – session with inactive deep brain stimulation. (For interpretation of the references to colour in this figure legend, the reader is referred to the web version of this article.)

Out of the 20 enrolled DY subjects, 3 DY subjects failed to complete the full MRI protocol due to intolerable discomfort in the DBS OFF condition, leaving 17 DY subjects for the final analysis. No subjects were excluded due to poor fMRI signal quality, brain coverage or excessive framewise root-mean-squared head motion exceeding 1 voxel (see Supplementary Table 1). In keeping with our previously established semiquantitative quality control procedures to account for the signal dropouts in the vicinity of the implanted leads and looped subcutaneous extension cord, temporal signal-to-noise ratio (tSNR) was calculated over the selected parcels and low-thresholded at the level of 10. Contrary to our previous study (Filip et al., 2022), all the parcels met the previously required criterion of at least 90 % of eligible DY patients having tSNR in each parcel higher than 10, so no parcels were excluded due to low quality of signal.

However, in 5 subjects, the chosen stimulation settings did not provide upper and/or lower GPi area stimulation bilaterally. In 4 subjects, rsfMRI acquisition was available with bilateral stimulation only in the upper GPi area – the other rsfMRI acquisition was with “mixed positions”, where only one of the active stimulation contacts was in the lower GPi area, whereas the contralateral contact was also positioned in the upper GPi area. In 1 subject, the situation was reversed – one rsfMRI acquisition with bilateral lower GPi area stimulation was available, but the other rsfMRI acquisition was performed with one active contact in the upper GPi area and one contact in the lower GPi area (clearly below the GPi border as reconstructed using Lead-DBS). In the respective conditions of these “problematic” subjects, the data associated with the correct stimulation position in the “mixed position” session were mirrored (i.e. from the cerebral hemisphere contralateral to the correct stimulation position and ipsilateral cerebellar hemispheres) to replace the data derived from the incorrect stimulation position, to avoid fully excluding these subjects from the main analysis. Nonetheless, an additional, supplementary analysis was performed with only 12 non-problematic DY subjects.

In the last step, FSLNets was utilised to generate partial correlation matrices over this predefined sensorimotor network, with regularisation using L2-norm ridge regression, followed by the calculation of eigenvector centrality (EC) with the Brain Connectivity Toolbox (Rubinov and Sporns, 2010). Resting state physiological fluctuation amplitude (RSFA) was calculated using the AFNI package (Kannurpatti and Biswal, 2008, Cox, 1996). Subject-specific averages of these two parameters for the whole sensorimotor network were calculated as well.

### 2.5 Statistical analysis

Demographic and clinical information in all eligible subjects was summarised using descriptive statistics (see Table 1). Inter-group differences between HC and DY patients were evaluated using Fisher’s test for sex and Wilcoxon rank sum test for age; Wilcoxon signed-rank test was used for continuous variables in the comparison between lower and upper GPi area sessions (stimulation amplitude, impedance, total electrical energy delivered, VTA in GPi and sensorimotor part of GPi, respective clinical scores and clinical improvement). P values were False Discovery Rate (FDR) adjusted across the 11 considered comparisons (Benjamini and Hochberg, 1995).

**Table 1:** Basic demographic and clinical data. Only eligible subjects (not excluded in previous quality control steps) are considered in this table.

Demographic and clinical information	Dystonia patients	Healthy controls	p value(FDR)
Sex (M/F)	9/8	10/7	>0.50
Age	54 [25–76]	58 [26–81]	>0.50
**Axis I**			
Age of onset (years)	35 [2–58]		
Disease duration (years) – only available in 16 subjects	18 [9–40]		
Body distribution [cervical / generalised]	10 / 7		
Associated features [none / parkinsonism / other]	16 / 1 / 0		
**Axis II**			
Neurodegeneration / Static CNS lesions / None	0 / 0 / 17		
Inherited / Acquired / Idiopathic sporadic	0 / 0 / 17		
**Medication**			
Anticholinergics / Baclofen / Antiepileptic drugs	2 / 0 / 2		
Benzodiazepines / Antidepressants	7 / 7		
L-dopa / Dopamine agonist	1 / 1		
**DBS-related information**	**Lower GPi area**	**Upper GPi area**	
Stimulator type [Kinetra / RC Activa]	2 / 15		
Lead type [Medtronic 3389]	17		
Time since implantation (years)	6.1 [2.1–16.6]		
Clinical DBS response as evaluated by the attending physicianABCD[non-responder / partial responder / responder]	0 / 1 / 16		
Clinical stimulation mode [monopolar / bipolar / interleaved]	13 / 1 / 3		
Constant voltage / constant current mode	5 / 12		
Voltage (V) – bilateral average	1.5 [1.2–2.0]	1.6 [0.4–1.8]	>0.50
Current (mA) – bilateral average	1.9 [0.5–3.1]	1.8 [1.3–2.6]	>0.50
Pulse width (us)	210 [75–390]		
Frequency (Hz)	130 [50–130]		
Impedance (Ω) – only available in 16 subjects	930.0 [394.0–1667.0]	948.5 [514.5–1533.5]	>0.50
Total electrical energy delivered (µW) – only available in 16 subjects	132.9 [13.8–675.2]	139.8 [32.5–314.9]	>0.50
Volume of tissue activated in GPi [ml]	10.3 [1.8–45.3]	12.1 [0.3–36.2]	>0.50
Volume of tissue activated in sensorimotor part of GPi [ml]	5.0 [1.4–32.4]	5.3 [0.2–16.3]	>0.50

**Clinical and subjective effect of DBS**	**Lower GPi area**	**Upper GPi area**	

**TWSTRS (in 10 subjects with cervical distribution)**			
Before DBS implantation	24.0 [18.0–47.0]		
DBS-ON lower and upper GPi area	12.5 [2.0–23.0]	11.0 [5.0–26.0]	>0.50
DBS-OFF	16.5 [9.0–30.0]		
**BFMDS (in 7 subjects with generalized distribution)**			
Before DBS implantation – only available in 6 subjects	17.0 [0.0–50.5]		
DBS-ON lower and upper GPi area	20.0 [6.0–73.0]	20.0 [7.0–49.0]	>0.50
DBS-OFF	28.0 [7.0–49.0]		
**Improvement with DBS**			
Relevant scale DBS-ON (lower and upper GPi area) vs DBS OFF (%)	25.0 [-49.0–87.5]	25.0 [-80.0–70.6]	>0.50
Relevant scale – best effect [lower / upper / no difference]	9 / 6 / 2		
Subjective – best effect [lower/upper/no difference/OFF]	5 / 4 / 6 / 2		
Subjective – worst effect [lower/upper/ no difference/OFF]	2 / 2 / 6 / 7		

Continuous data displayed as median [range], categorical variables as numbers of subjects in the relevant group. P value corresponds to the statistical test comparing relevant groups (Fisher’s exact test for categorical variables, Wilcoxon rank sum test for continuous variables in non-paired fashion [age comparison between healthy controls and dystonia patients], and Wilcoxon signed rank test for paired continuous variables) after false discovery rate correction. Abbreviations: FDR – false discovery rate; M – male; F – female; CNS – central nervous system; DBS – deep brain stimulation; GPi – internal globus pallidus; V – Volt, mA – milliampere; us – microsecond; Hz – Hertz; Ω − Ohm; TWSTRS – Toronto Western Spasmodic Torticollis Rating Scale; BFMDS – Burke-Fahn-Marsden Dystonia Scale.

The comparisons between HC and DY patients for parcellated CIFTI maps were based on general linear models (GLM), with subject group as fixed factor, and sex and age as covariates of non-interest. Repeated-measures GLMs were utilised for the comparison between lower and upper GPi area stimulation sessions, again separately for the whole sensorimotor network averages and for the parcellated CIFTI maps, with the main interaction analysis being based on the comparison of (lower GPi area stimulation – DBS OFF) vs (upper GPi area stimulation – DBS OFF). This model was run twice, once for the full 17 dystonia patients and once for 12 dystonia patients without the need of signal mirroring from the other hemispheres (see above). Lastly, a validatory supplementary GLM correlating the percentual clinical improvement with EC and RSFA was constructed, separately for the lower and upper GPi area stimulation. Permutation-based non-parametric analysis as implemented in the Permutation Analysis of Linear Models package (Winkler et al., 2014) was utilised, with 10,000 permutations, threshold-free cluster enhancement (TFCE) based on anatomical proximity of individual parcels, cortical clustering threshold of 2 (to exclude singleton cortical parcels). First- (over parcels) and second-level (over modalities) FDR correction (Benjamini and Hochberg, 1995) in each GLM model was implemented, with predetermined alpha of 0.05 considered statistically significant.

## 3 Results

Table 1 provides basic demographic and clinical information. 10 patients had idiopathic cervical dystonia, 7 patients generalised dystonia, one of them with mild parkinsonism symptoms. The supplementary analysis, which excluded DY subjects with contralateral cerebral and ipsilateral cerebellar hemispheres mirrored due to the unsuitability of one of the stimulation contacts, was based on 9 and 3 cervical and generalised dystonia cases, respectively. No statistically significant inter-group differences were detected.

### 3.1 Lead position

DBS-lead placement and active contacts in the lower and the upper GPi area stimulation conditions are depicted in the Supplementary Figure 2 (in blue and red colour, respectively). There was only a mild numeric difference in VTA in GPi between these two stimulation settings, not reaching statistical significance.

**Fig. 2:**
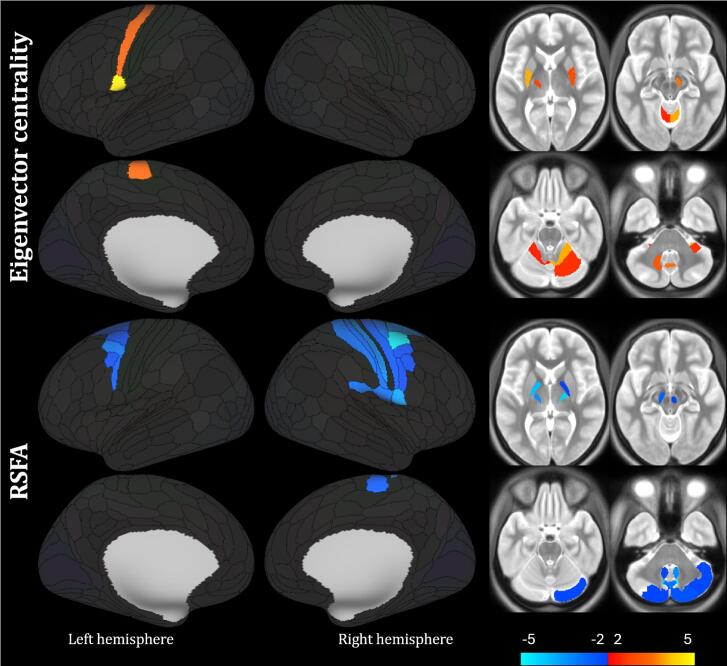
Main results for the interaction analysis of the effect of the stimulation in the lower and upper GPi part vs DBS OFF state in dystonia patients for RSFA and eigenvector centrality. Alpha of 0.05, false discovery rate corrected. Red-yellow scale marks showing the (lower GPi part vs DBS OFF) > (upper GPi part vs DBS OFF) contrast; blue scale labels the reverse contrast; values correspond to the T statistic. Subcortical structures shown in 4 slices z = 4, −10, −24, −38 (MNI coordinate system). Laterality convention where the right side of the figure corresponds to the right side of the brain is used. See Table 2 for further anatomical and statistical information on significant regions. For the full information on individual parcels utilised as nodes, see (Glasser et al., 2016). Abbreviations: RSFA – resting state physiological fluctuation amplitude. (For interpretation of the references to colour in this figure legend, the reader is referred to the web version of this article.)

### 3.2 Comparison between HC and DY

Supplementary Table 2 and Supplementary figure 3 depict the comparison between HC and DY DBS OFF condition, showing significantly higher RSFA and lower EC in DY patients over large areas of the sensorimotor network.

### 3.3 Lower and upper GPi area stimulation – Global effect over the whole sensorimotor network

Table 2 and Fig. 1 show the average RSFA and EC values in 3 DBS settings and in HC, including their statistical comparisons. The comparison between HC and DY patients revealed statistically significant differences in both RSFA (all three DBS settings) and EC (only in the contrast HC vs. DBS OFF and HC vs. upper GPi area stimulation; the contrast HC vs lower GPi area stimulation yielded no statistically significant results). However, no significant differences were detected in the pair-wise comparisons of RSFA and EC averages over the whole sensorimotor network between lower and upper GPi area stimulation (see Table 2). Similarly, the interaction analysis (lower GPi area stimulation – DBS OFF) vs (upper GPi area stimulation – DBS OFF) yielded no statistically significant differences (not presented in the Table 2; FDR corrected p-values 0.163 and 0.120 for RSFA and EC, respectively). When comparing the individual EC and RSFA values for the three implemented DBS settings, 13 subjects had the lowest RSFA (intra-individual value closest to the level in HC) in the lower GPi area stimulation and 4 subjects in the DBS OFF state. For EC, it was 9, 6 and 2 subjects with highest EC in the lower GPi area stimulation, upper GPi area stimulation and DBS OFF, respectively (see Supplementary Table 3 for the complete dataset, including individual subject values).

**Table 2:** Comparison of full sensorimotor network parameters between healthy controls and dystonia patients with DBS ON in the lower GPi part, upper GPi part and in DBS OFF condition.

Parameters presented as average [standard deviation] of the whole sensorimotor network for RSFA (grey row in the upper part of the table) and eigenvector centrality (white row in the upper part of the table). The lower part of the table contains False Discovery Rate corrected p values for comparisons between individual groups, lower triangle (grey) for RSFA, upper triangle (white) for eigenvector centrality (two-sample T-test for the comparison between healthy controls and individual dystonia acquisitions; paired T-test for the comparison of acquisitions with various DBS settings within dystonia group). Statistically significant results marked in bold italics with an asterisk. Abbreviations: Dyst – dystonia; DBS-ON– session with active deep brain stimulation; DBS-OFF – session with inactive deep brain stimulation; GPi – internal globus pallidus.

### 3.4 Lower and upper GPi area stimulation – Parcellated analysis for localised effects

Table 3 and Fig. 2 provide the main results of the interaction analysis comparing the lower and upper GPi area stimulation after subtracting the individual DBS OFF levels, showing significant differences in cortical, subcortical and cerebellar areas in the direction contrary to the one detected in the comparison between HC and DY DBS OFF session, even though the number and size of significant clusters is lower. Nodes of key importance in the pathophysiological models of dystonia – sensorimotor cortex, striatum, thalamus and cerebellum – yielded statistically significant results for both EC and RSFA. Furthermore, differences were detected in numerous smaller subcortical grey matter structures as substantia nigra and red nucleus, albeit with strong lateralisation, pointing to the direct or indirect effect of GPi DBS even in these areas. The supplementary analysis excluding the data from DY subjects with contralateral cerebral and ipsilateral cerebellar hemispheres mirrored due to the unsuitability of one of the stimulation contacts provided very similar results, even though with less conservative statistical thresholding (see Supplementary Table 4 and Supplementary figure 4).

**Table 3:** Main results for the interaction analysis of the effect of lower and upper stimulation position vs DBS OFF state in dystonia patients for eigenvector centrality and RSFA.

	Side and anatomical area		#	Healthy controls	Dystonia DBS ON Lower GPi	Dystonia DBS ON Upper GPi	Dystonia DBS OFF	T value	p val(FDR)
Eigenvector centrality lower vs OFF > upper vs OFF	L	Posterior Operculum, Somatosensory and Motor Cortex	2	*0.126 [0.030]*	0.113 [0.036]	0.089 [0.050]	0.107 [0.058]	−4.700	0.003
	L	Putamen (sensorimotor)	1	*0.070 [0.031]*	0.066 [0.037]	0.045 [0.028]	0.051 [0.026]	−4.032	0.003
	R	Cerebellum (lobule V, VI)	2	*0.116 [0.038]*	0.128 [0.060]	0.106 [0.047]	0.085 [0.053]	−4.005	0.003
	C	Cerebellum (vermis VIIIb)	1	*0.085 [0.058]*	0.089 [0.071]	0.052 [0.035]	0.085 [0.124]	−3.547	0.006
	R	Substantia nigra	1	*0.044 [0.038]*	0.046 [0.040]	0.030 [0.015]	0.042 [0.031]	−3.563	0.006
	R	Putamen (sensorimotor)	1	*0.076 [0.028]*	0.084 [0.029]	0.069 [0.037]	0.060 [0.033]	−2.943	0.007
	L	Cerebellum (lobule V, dentate)	2	*0.075 [0.026]*	0.084 [0.039]	0.068 [0.029]	0.058 [0.026]	−3.150	0.020
	L	Thalamus (premotor)	1	*0.044 [0.025]*	0.042 [0.032]	0.029 [0.018]	0.032 [0.028]	−2.746	0.027

RSFA upper vs OFF > lower vs OFF	R	Premotor Cortex, Somatosensory and Motor Cortex, Posterior Operculum	13	*2.304 [0.573]*	3.942 [0.804]	4.397 [0.931]	5.027 [1.333]	4.909	0.001
	L	Cerebellum (crus II, lobule VIIIa, VIIIb, IX)	4	*1.734 [0.465]*	3.247 [1.083]	4.131 [1.783]	4.194 [1.636]	4.519	0.001
	R	Cerebellum (crus I, II, lobule VIIIa, VIIIb, IX)	5	*1.932 [0.441]*	3.301 [0.792]	4.232 [1.594]	4.170 [1.280]	5.135	0.001
	R	Thalamus (premotor)	1	*1.759 [0.275]*	2.893 [0.416]	3.167 [0.361]	3.260 [0.723]	4.349	0.001
	C	Cerebellum (vermis VIIIa)	1	*2.208 [0.557]*	4.704 [1.618]	5.566 [2.252]	5.600 [3.059]	2.924	0.003
	L	Pallidum (external)	1	*1.569 [0.242]*	3.195 [0.891]	3.615 [0.923]	3.165 [0.928]	4.046	0.003
	L	Thalamus (premotor)	1	*1.848 [0.401]*	3.202 [0.573]	3.484 [0.644]	3.554 [0.680]	3.341	0.003
	L	Premotor Cortex	5	*2.433 [0.667]*	4.261 [0.922]	4.665 [1.035]	5.035 [1.391]	2.873	0.013
	L	Caudate (sensorimotor)	1	*2.930 [0.961]*	4.903 [1.768]	5.534 [1.433]	5.501 [1.943]	2.822	0.015
	R	Caudate (sensorimotor)	1	*3.029 [0.588]*	4.789 [1.138]	5.472 [1.810]	5.914 [2.327]	2.341	0.031
	R	Pallidum (external)	1	*1.635 [0.270]*	3.176 [1.065]	3.408 [0.947]	3.242 [0.933]	2.005	0.038
	R	Red nucleus	1	*3.130 [0.609]*	5.966 [2.311]	6.645 [2.277]	6.562 [2.855]	2.056	0.039
	L	Substantia nigra	1	*2.401 [0.685]*	4.645 [1.981]	5.576 [2.391]	4.886 [2.001]	2.519	0.042

Data reported as clusters, with cortical anatomical localisation based on 22 main cortical segments and parcellation as defined by (Glasser et al., 2016), number of parcellation regions of interest contained in each cluster (column #), average and standard deviation for the stated clusters separately for healthy controls in italics for comparison purposes, and dystonia group with DBS active in lower GPi part, in upper GPI part and DBS off, followed by the T statistic and p value. Alpha of 0.05, False Discovery Rate corrected was implemented. Individual entries sorted in descending order based on the effect size. See also Fig. 2. Abbreviations: DBS – deep brain stimulation; GPi – internal globus pallidus; T stat – T statistic; FDR – False Discovery Rate; L – left; R – right; C – central; RSFA – resting state physiological fluctuation amplitude.

### 3.5 Correlation between clinical improvement and rs-fMRI metric

The correlation between rs-fMRI metrics and the DBS-related clinical improvement provided positive results for EC in the lower GPi area stimulation session. Moreover, clinical improvement was inversely related to the effect of lower GPi area stimulation on connectivity of the posterior cerebellar lobe. Interestingly, these cerebellar areas were different than the parts detected in the comparison between DY and HC subjects or lower and upper GPi area stimulation (see Supplementary Table 5 and Supplementary Figure 5). This model failed to yield any statistically significant results for EC in upper GPi area stimulation session and for RSFA.

## 4 Discussion

The presented single-blinded, random-order study with patient blinding against the stimulation condition and cross-sectional validation against HC builds upon the previous body of research into the effect of DBS in dystonia, with the intent to evaluate the rs-fMRI signature of the hypothesised sweet spot lying in the lower part of the GPi or underneath it. In accordance with our previous findings, GPi DBS has been found to bring not only functional connectivity (Filip et al., 2022), but also local activity reported in a study by another team (Loh et al., 2022) to levels closer to the healthy controls. Importantly though, this effect was much more pronounced when stimulating in the lower GPi area or beneath it than in slightly higher positions.

Lower level of functional connectivity in the sensorimotor network in DY patients detected in our cohort is generally in accord with the previous body of literature (Filip et al., 2022, Delnooz et al., 2012, Delnooz et al., 2013), even though there seems to be a divergent pattern in other regions as executive control networks where dystonia patients have been shown to exhibit increased functional connectivity (Delnooz et al., 2013, Haslinger et al., 2017). Another factor contributing to the general variability of connectivity analyses stems from the wide spectrum of connectivity measures available, ranging from simple averages of voxel-wise correlation coefficients to more complex measures presumably of far higher biological relevance (Marrelec et al., 2005). In addition to the cortical surface-based analysis with multimodal surface matching superior to simple analyses in MNI space (Robinson et al., 2018), combined with complex parcellation approach ensuring effective smoothing over homogeneous areas and mitigating the multiple-comparison problem of voxel-wise analyses, the presented study provides three levels of further optimisation: 1) connectivity is based on partial correlations as much more viable estimates of conditional dependence between the activities of two regions, as the effect of other regions has been regressed out; 2) eigenvector centrality was chosen as a connectivity measure hypothesised to surpass simpler metrics based on its higher biological relevance (Lohmann et al., 2010); and 3) connectivity was calculated only over a pre-selected network of interest, thereby removing spurious influences of other regions with uncertain relevance. Nonetheless, despite all the alterations to our analysis approach when compared to our previous study on GPi DBS effects in dystonia (Filip et al., 2022), the main areas, direction of effect and correlation with DBS-elicited clinical improvement (see Supplementary Fig. 5 and Supplementary Table 5 were similar to the previous study, providing further backing to our results.

As for local activity in general, where the presented study employed RSFA as a viable proxy with documented biological pertinency (Kannurpatti and Biswal, 2008), regional variability comparable to that of functional connectivity has been observed in similar parameters as amplitude of low frequency fluctuations or in metabolic studies. Increased local activity in brain regions relevant for sensorimotor functions has been reported, but available literature is far from homogeneous in this area (Feng et al., 2021, Zhou et al., 2013, Yang et al., 2013). Fluorodeoxyglucose positron emission tomography studies have also shown a general activity increase in relevant sensorimotor regions in several dystonia types (Galardi et al., 1996, Magyar-Lehmann et al., 1997, Suzuki et al., 2007). Most importantly though, optimal GPi DBS settings have been reported to decrease the local activity measured using amplitude of low-frequency fluctuations in the sensorimotor cortex when compared to non-optimal GPi DBS settings or DBS OFF condition (Loh et al., 2022), even though other areas experienced local activity increases in this paradigm.

For both RSFA and EC, the direction of change elicited by DBS in our study – increase for EC and decrease for RSFA – was towards the state detected in healthy controls. This is well in accord with the general understanding of DBS impact also in other diseases and stimulation areas, e.g. in DBS of the subthalamic nucleus in Parkinson’s disease (Jech and Mueller, 2022, Filip et al., 2024). Importantly, this effect was more significant during the stimulation in the lower GPi area encompassing the subpallidal white matter tracts (see Fig. 1, Fig. 2, Table 3). This finding is in full concordance with the probabilistic map for stimulation induced motor benefits supporting the ventro-posterior internal globus pallidus and the subpallidal white matter in the proximity as the purported “sweet spot” for DBS effects (Reich et al., 2019). Anatomical characteristics of this area exhibit substantial complexity. It contains not only white matter of several intertwining tracts, but also substantia incerta, specifically basal nucleus of Meynert, a cholinergic node previously implicated as cortical plasticity modulator and even speculated as a DBS target in different neurodegenerative diseases (Nazmuddin et al., 2021). Neuronal depletion of this area has been implicated in both Alzheimer’s and Parkinson’s disease, with associated neuropsychiatric and cognitive deficits (Liu et al., 2015). Furthermore, it boasts vast connectivity to important cortical regions, including prefrontal, parietal, temporal and also cingulate cortex (Hepp et al., 2017), indicating large-network effects of stimulating this region. Both fMRI metrics employed in the presented study detected stimulation-related changes mostly in the sensorimotor cortex, striatum, thalamus and the cerebellum, but also in smaller subcortical grey matter structures as red nucleus and substantia nigra, although with strong and pathophysiologically dubious lateralisation, which probably stemmed from statistical thresholding and low number of included subjects. Moreover, GPi DBS led to the decrease of the spread of inter-individual variability of RSFA as visible in the Fig. 1 and standard deviations in Table 2, while for EC, there was generally a shift of the group average towards the level seen in healthy controls without any change of spread. This finding itself is an important notion considering the general clinical variability of dystonia and also the substantial variability of DBS effect in DY patients in our cohort, as visible in subjective and objective evaluations of effects and also individual directions of rs-fMRI parameter changes depicted by dotted lines connecting values of the three DBS states in each DY patient in Fig. 1. Nonetheless, the outputs must be seen in the light of generally high inter- and intra-individual variability of the majority of rs-fMRI metrics as evidenced by non-negligible spread of EC values even in the HC group. Further studies should consider implementing the combination of structural connectome based on diffusion-weighted imaging with rs-fMRI metrics. Although seemingly not fully compatible and providing disparate indications, their combination may yet help to overcome the disadvantages of both methods (Lv and Calamante, 2023).

Several limitations must be considered in the context of this study. The short time between the changes of DBS settings was definitely not sufficient to elicit all the eventual effects of GPi DBS as clinically seen in dystonia. However, a study design requiring patients to spend several weeks with potentially suboptimal DBS settings substantially affecting their quality of life would be non-ethical in the least. A side note of interest may be the fact that one of the study participants felt a clear subjective improvement with the lower GPi area stimulation which had not been previously used in her chronic settings and as of the publication of this manuscript, her DBS settings still utilise the most ventral contacts despite them being positioned clearly under GPi itself. Secondly, the presence of artifacts related to DBS hardware in the rs-fMRI data is an inherent confounder to this analysis. This issue is also hampering attempts at multimodal studies into the effects of DBS in real-world settings due to substantial risks associated with longer acquisition protocols and/or more advanced, semiquantitative MRI methods. Given the poor record of rs-fMRI in general reproducibility of studies (Griffanti et al., 2016) and its complicated inferences on the underlying neural activity (Winder et al., 2017), it is imperative to develop and employ further methods enabling relevant data acquisition under these constraints. Thirdly, the enrolled patient cohort consists of both segmental and generalised dystonia cases, which may present a substantial confounder given the previously reported associated differences in the clinical effect (Volkmann et al., 2012). Unfortunately, the sample size does not provide sufficient statistical power for the comparison between rs-fMRI effects of GPi DBS based on these phenotypical differences. Further studies should consider combining patient pools from multiple clinical sites to enable more detailed analyses. Lastly, we definitely agree with previous conclusions (Reich et al., 2019) advising against overzealous clinical implementation of the area under GPi as the future primary target of DBS implantation in dystonia patients. Further studies will be necessary to substantiate these findings, even though the intentional planning for at least one DBS lead contact positioned in the lower part of GPi and/or slightly under it, similarly to the majority of patients presented in this study, is for consideration.

## 5 Conclusion

While building upon and confirming the results of two previous studies on GPi DBS as a modality restoring both sensorimotor network connectivity and local activity in dystonia towards the state seen in healthy controls, our results also indicated that this effect was more pronounced when stimulating in the lower GPi area, including the subpallidal white matter. Furthermore, the immediate nature of fMRI signature change, now repeatedly shown in multiple cohorts, opens further possibilities for the integration of this method into clinical DBS programming.

## Funding statement

Support was provided by the National Institute for Neurological Research, Czech Republic, Programme EXCELES (ID project No. LX22NPO5107), General University Hospital in Prague (MH CZ-DRO-VFN64165), the European Union's Horizon 2020 research and innovation programme under the EJP RD COFUND-EJP N° 825,575 − EurDyscover, and the Brain Dynamics (grant number CZ.02.01.01/00/22_008/0004643).

**Code availability:** not applicable.

Authors’ contributions

P.F. participated in the design of the study protocol and data analysis approach, performed the data analysis, created the figures and tables and wrote the manuscript. A.L. managed the MRI data acquisition and edited the manuscript. D.K., F.R., D.U., D.N. and K.M. edited the manuscript. J.K. handled MRI data acquisition and edited the manuscript. R.J. ensured the funding for the study, participated in the design of the study protocol, and edited the manuscript.

**Ethics approval:** The study protocol was approved by the ethics committee of the General University Hospital in Prague, Czech Republic.

**Consent to participate:** Each subject provided a written informed consent in accordance with the Declaration of Helsinki

## CRediT authorship contribution statement

**Pavel Filip:** Writing – original draft, Software, Methodology, Formal analysis, Conceptualization. **Andrej Lasica:** Resources, Project administration, Data curation. **Dimitra Kiakou:** Writing – review & editing. **Karsten Mueller:** Writing – review & editing. **Jiří Keller:** Writing – review & editing. **Dušan Urgošík:** Writing – review & editing. **Daniel Novák:** Writing – review & editing. **Robert Jech:** Writing – review & editing, Supervision, Funding acquisition, Conceptualization.

## Declaration of Competing Interest

The authors declare that they have no known competing financial interests or personal relationships that could have appeared to influence the work reported in this paper.

## Data Availability

Data will be made available on request.
