# Supplementary material for: Sweet spot for resting-state functional MRI effect of deep brain stimulation in dystonia lies in the lower pallidal area
Source: Neuroimage Clin. 2025 Feb 5;45:103750. doi: 10.1016/j.nicl.2025.103750 (PMC11889665; doi:10.1016/j.nicl.2025.103750)
Supplement: Supplementary Data 1 [file mmc1.docx]

# Supplementary methods

## Subjects

20 DY patients (10 females, median age [range] of 53 [24–75] years) managed with chronic GPi DBS therapy were enrolled into this study. The diagnosis of dystonia has been confirmed by a tertiary-care movement disorders centre specialist in accordance with the relevant diagnostic criteria [1]. Inclusion criteria were as follows: the presence and/or history of primary dystonia without head tremor (to avoid MRI artifacts), active bilateral GPi DBS as a part of dystonia management for at least 6 months before the participation in this study, stable DBS parameters for at least 1 month before the participation in this study. General exclusion criteria were as follows: neurological and/or psychiatric disorder other than dystonia, general contraindications to MRI examination, a non-negligible vascular or space occupying central nervous system lesion other than the implanted DBS system. Clinical data recorded within this study included the following: neurological status based on the referenced dystonia classification (axis I – clinical characteristics [age of onset, body distribution, temporal pattern and associated features] and axis II – aetiology [nervous system pathology, inherited/acquired status] and current medication [1]. The clinical condition was evaluated using the motor score subsections of either Burke-Fahn-Marsden Dystonia Scale (BFMDS) [2], or Toronto Western Spasmodic Torticollis Rating Scale (TWSTRS) [3] based on the clinical presentation (generalised or cervical dystonia, respectively), at four time points – before DBS implantation, before two DBS-ON rs-fMRI acquisitions and before DBS-OFF acquisition (see the Imaging Protocol in the following text). While BFMDS evaluates dystonic symptoms based on movement and disability subscales in nine body regions, TWSTRS provides information only on cervical symptomatology. And lastly, information related to the DBS system was recorded (hardware information, time since the implantation, clinical response to DBS according to the attending neurologist and stimulation settings in the two DBS programs utilised in the study [stimulation mode (constant current or constant voltage), active contacts, amplitude, pulse width, frequency and therapy impedance]). The clinical effect of DBS was calculated as the ratio of the difference between the clinical scores (BFMDS or TWSTRS according to the clinical presentation) at relevant timepoints or DBS settings (positive values corresponded to clinical improvement and vice versa).

Healthy controls (HC) (10 females, median age [range] of 58 [26-81]) were selected from the pool of previously acquired subjects [4] utilising Matchit r-library based on age and sex of dystonia patients [5].

Every subject provided their written informed consent form in accordance with the Declaration of Helsinki. The study protocol was approved by the ethics committee of the General University Hospital in Prague.

Imaging protocol

The investigational MRI protocol acquired using a 1.5 Tesla Siemens Avanto System (Siemens, Erlangen, Germany) included a T1-weighted (T1w) structural scan and rs-fMRI session(s) (one in HC and three in DY patients, see below for details). It followed the previously published technical recommendations for the potential hazards associated with intracerebral electrodes [6]. The following acquisition parameters were utilised:

- T1w scan – magnetisation-prepared rapid gradient echoes (MPRAGE), 1.0-mm isotropic resolution, repetition time (TR) 2,140 ms, inversion time (TI) 1,100 ms, echo time (TE) 3.93 ms, flip angle (FA) 15°.
- rs-fMRI – gradient-recalled echo (GRE) echo-planar imaging (EPI) sequence, in-plane resolution 3x3 mm^2^ (image matrix of 64x64 pixels), slice thickness 3 mm, interslice gap 1 mm, 31 slices to cover the whole brain including the cerebellum, TR 3,000 ms, TE 51 ms, FA 90°, 200 volumes (acquisition time 10 minutes), anterior-posterior phase encoding polarity. The subjects were asked to lie motionlessly with the eyes open fixated on a cross in the middle of their visual field. An MRI compatible 12M camera (MRC Systems, Heidelberg, Germany) was used to monitor wakefulness.

While HC underwent only one rs-fMRI acquisition, DY patients underwent three rs-fMRI acquisitions in random order across the subjects, with patients blinded to the utilised DBS settings (the investigator was not blinded): A) DBS switched off (DBS-OFF session); B) active DBS utilising the stimulation parameters determined by the attending physician; C) active DBS with different contact settings to the previous condition, so that either the previous or this session used the lowest possible stimulation contacts. Pulse width and frequency were maintained, and the highest tolerable stimulation amplitude was set. Individual rs-fMRI acquisitions were separated by at least 20 minutes.

No medication alterations or discontinuations were introduced as a part of the MRI protocol.

DBS electrode position analysis

The position of DBS electrode was analysed using Lead-DBS software (version 2.5.2) with the enhanced workflow [7,8]. T1w scans from the investigational MRI protocol as described above were co-registered, including brain shift correction [9], to the pre-operative T1w scans, which had been acquired separately as part of the routine clinical work-up for DBS patients. In the following step, the investigational T1w scan was warped to the Montreal Neurological Institute (MNI) space based on the warp field determined using co-registration of the preoperative T1w scan to MNI space with diffeomorphic registration algorithm (Advanced Normalization Tools (Avants et al., 2008)), including the subsequent subcortical refinement [8]. The last preparatory step included automatic pre-localisation of electrode trajectories with subsequent manual refinement and delineation of volume of tissue activated (VTA) based on the estimated electric fields around the active contacts (for both the settings implemented in this study) using finite element approach, electric field gradient threshold of 0.2 V/mm [10]. Furthermore, simple overlap of VTA and the whole GPi and sensorimotor part of GPi based on the DISTAL atlas [11] was calculated.

Structural and functional MRI data analysis

The processing pipeline was based on the Human Connectome Project (HCP) pipeline [12], specifically its “legacy” version, which requires only a T1-weighted full-brain scan. Briefly, it consists of rigid-body alignment to MNI space (1-mm isotropic voxel template), initial brain mask extraction based on non-linear (FNIRT as implemented in FSL) registration of the T1w image to the MNI template, followed by CUDA (Computer Unified Device Architecture)-enabled version of FreeSurfer 6.0. The output of FreeSurfer was used for the final MNI space non-linear warp (specifically the brain mask generated by FreeSurfer segmentation) and registration to the Conte69 population-average surfaces (specifically native-mesh surfaces provided by FreeSurfer) and the combined 2-mm standard Connectivity Informatics Technology Initiative (CIFTI) grayordinate space to be utilised for the following rs-fMRI pipeline.

rs-fMRI processing also followed the HCP pipeline and included slice timing correction, spatial realignment to correct for subject motion (3D rigid-body motion correction of each frame of the timeseries to the first scan using FSL 6.0 MCFLIRT), co-registeration to the structural T1w scan to be combined with the 2-mm isovoxel MNI-space warp from the previous step and mapping to the standard CIFTI grayordinate space utilising partial volume weighted ribbon-constrained volume to surface mapping algorithm (excluding the voxels with locally high temporal coefficient of variation) with 2-mm full-width at half maximum surface and subcortical volume smoothing [12]. The next steps utilised the HCP rs-fMRI pipeline [13] – MELODIC independent component analysis and FIX algorithm for the identification of artefactual components with subsequent “non-aggressive” regression of the contribution or artefactual MELODIC components and subject motion [14].

A trained operator (P.F.) visually evaluated the adequacy of FreeSurfer surface reconstruction and segmentation (inaccuracies corrected manually in 3 DY and 2 HC subjects), co-registration and motion correction step and signal dropouts.

Afterwards, processed rs-fMRI data were parcellated with a combination of HCP cortical parcellation (180 parcels per hemisphere) [15], Oxford thalamic connectivity atlas [16], Oxford-GSK-Imanova connectivity striatal atlas [17] divided into the respective caudate and putamen subsegments based on FreeSurfer subcortical segmentation, probabilistic structural cerebellar atlas [18], and ATAG atlas [19] for external and internal pallidum, red nucleus, substantia nigra and subthalamic nucleus. Only parcels of sensory-motor areas were considered in the further analysis (see Supplementary figure 1): somatosensory and motor cortex and premotor cortex [15], motor-function-related cerebellar structures (lobules I-IV, V, VI, VIIIa, VIIIb,, IX, crus I and II; dentate) [20], sensory-motor parts of caudate and putamen, primary motor, premotor and sensory parts of thalamus, external and internal globus pallidus, red nucleus, substantia nigra and subthalamic nucleus; yielding 76 parcels altogether.

Out of the 20 enrolled DY subjects, 3 DY subjects failed to complete the full MRI protocol due to intolerable discomfort in the DBS OFF condition, leaving 17 DY subjects for the final analysis. No subjects were excluded due to poor fMRI signal quality, brain coverage or excessive framewise root-mean-squared head motion exceeding 1 voxel (see Supplementary table 1). In keeping with our previously established semiquantitative quality control procedures to account for the signal dropouts in the vicinity of the implanted leads and looped subcutaneous extension cord, temporal signal-to-noise ratio (tSNR) was calculated over the selected parcels and low-thresholded at the level of 10. Contrary to our previous study, all the parcels met the previously required criterion of at least 90% of eligible DY patients having tSNR in each parcel higher than 10, so no parcels were excluded due to low quality of signal.

However, in 5 subjects, the chosen stimulation settings did not provide upper and/or lower GPi area stimulation bilaterally. In 4 subjects, rsfMRI acquisition was available with bilateral stimulation only in the upper GPi area – the other rsfMRI acquisition was with “mixed positions”, where only one of the active stimulation contacts was in the lower GPi area, whereas the contralateral contact was also positioned in the upper GPi area. In 1 subject, the situation was reversed – one rsfMRI acquisition with bilateral lower GPi area stimulation was available, but the other rsfMRI acquisition was performed with one active contact in the upper GPi area and one contact in the lower GPi area (clearly below the GPi border as reconstructed using Lead-DBS). In the respective conditions of these “problematic” subjects, the data associated with the correct stimulation position in the “mixed position” session (i.e. from the cerebral hemisphere contralateral to the correct stimulation position and ipsilateral cerebellar hemispheres) were mirrored to replace the data derived from the incorrect stimulation position, to avoid fully excluding these subjects from the main analysis. Nonetheless, an additional, supplementary analysis was performed with only 12 non-problematic DY subjects.

In the last step, FSLNets was utilised to generate partial correlation matrices over this predefined sensorimotor network, with regularisation using L2-norm ridge regression, followed by the calculation of eigenvector centrality (EC) with the Brain Connectivity Toolbox [21]. Resting state physiological fluctuation amplitude (RSFA) was calculated using the AFNI package [22,23]. Subject-specific averages of these two parameters for the whole sensorimotor network were calculated as well.

## Statistical analysis

Demographic and clinical information in all eligible subjects was summarised using descriptive statistics (see Table 1). Inter-group differences between HC and DY patients were evaluated using Fisher’s test for sex and Wilcoxon rank sum test for age; Wilcoxon signed-rank test was used for continuous variables in the comparison between lower and upper GPi area sessions (stimulation amplitude, impedance, total electrical energy delivered, VTA in GPi and sensorimotor part of GPi, respective clinical scores and clinical improvement). P values were False Discovery Rate (FDR) adjusted across the 11 considered comparisons [24].

The comparisons between HC and DY patients for parcellated CIFTI maps were based on general linear models (GLM), with subject group as fixed factor, and sex and age as covariates of non-interest. Repeated-measures GLMs were utilised for the comparison between lower and upper GPi area stimulation sessions, again separately for the whole sensorimotor network averages and for the parcellated CIFTI maps, with the main interaction analysis being based on the comparison of (lower GPi area stimulation – DBS OFF) vs (upper GPi area stimulation – DBS OFF). This model was run twice, once for the full 17 dystonia patients and once for 12 dystonia patients without the need of signal mirroring from the other hemispheres (see above). Lastly, a validatory supplementary GLM correlating the percentual clinical improvement with EC and RSFA was constructed, separately for the lower and upper GPi area stimulation. Permutation-based non-parametric analysis as implemented in the Permutation Analysis of Linear Models package [25] was utilised, with 10,000 permutations, threshold-free cluster enhancement (TFCE) based on anatomical proximity of individual parcels, cortical clustering threshold of 2 (to exclude singleton cortical parcels). First- (over parcels) and second-level (over modalities) FDR correction [24] in each GLM model was implemented, with predetermined alpha of 0.05 considered statistically significant.

# References

[1] Albanese A, Bhatia K, Bressman SB, DeLong MR, Fahn S, Fung VSC, et al. Phenomenology and classification of dystonia: A consensus update. Movement Disorders 2013.

[2] Burke RE, Fahn S, Marsden CD, Bressman SB, Moskowitz C, Friedman J. Validity and reliability of a rating scale for the primary torsion dystonias. Neurology 1985;35:73–73.

[3] Consky ES, Basinski A, Belle L, Ranawaya R, Lang AE. The Toronto Western Spasmodic Torticollis Rating Scale (TWSTRS): assessment of validity and inter-rater reliability. Neurology 1990;40:445.

[4] Mueller K, Jech R, Hoskovcová M, Ulmanová O, Urgošík D, Vymazal J, et al. General and selective brain connectivity alterations in essential tremor: A resting state fMRI study. NeuroImage: Clinical 2017;16:468–76. https://doi.org/10.1016/j.nicl.2017.06.004.

[5] Ho D, Imai K, King G, Stuart EA. MatchIt: nonparametric preprocessing for parametric causal inference. Journal of Statistical Software 2011;42:1–28.

[6] Jech R, Urgošík D, Tintěra J, Nebuželskỳ A, Krásenskỳ J, Liščák R, et al. Functional magnetic resonance imaging during deep brain stimulation: a pilot study in four patients with Parkinson’s disease. Movement Disorders: Official Journal of the Movement Disorder Society 2001;16:1126–32.

[7] Horn A, Kühn AA. Lead-DBS: a toolbox for deep brain stimulation electrode localizations and visualizations. Neuroimage 2015;107:127–35.

[8] Horn A, Li N, Dembek TA, Kappel A, Boulay C, Ewert S, et al. Lead-DBS v2: Towards a comprehensive pipeline for deep brain stimulation imaging. Neuroimage 2019;184:293–316.

[9] Schönecker T, Kupsch A, Kühn AA, Schneider G-H, Hoffmann K-T. Automated optimization of subcortical cerebral MR imaging- atlas coregistration for improved postoperative electrode localization in deep brain stimulation. American Journal of Neuroradiology 2009;30:1914–21.

[10] Vasques X, Cif L, Hess O, Gavarini S, Mennessier G, Coubes P. Stereotactic model of the electrical distribution within the internal globus pallidus during deep brain stimulation. Journal of Computational Neuroscience 2009;26:109.

[11] Ewert S, Plettig P, Li N, Chakravarty MM, Collins DL, Herrington TM, et al. Toward defining deep brain stimulation targets in MNI space: a subcortical atlas based on multimodal MRI, histology and structural connectivity. Neuroimage 2018;170:271–82.

[12] Glasser MF, Sotiropoulos SN, Wilson JA, Coalson TS, Fischl B, Andersson JL, et al. The minimal preprocessing pipelines for the Human Connectome Project. Neuroimage 2013;80:105–24.

[13] Smith SM, Beckmann CF, Andersson J, Auerbach EJ, Bijsterbosch J, Douaud G, et al. Resting-state fMRI in the human connectome project. Neuroimage 2013;80:144–68.

[14] Salimi-Khorshidi G, Douaud G, Beckmann CF, Glasser MF, Griffanti L, Smith SM. Automatic denoising of functional MRI data: combining independent component analysis and hierarchical fusion of classifiers. Neuroimage 2014;90:449–68.

[15] Glasser MF, Coalson TS, Robinson EC, Hacker CD, Harwell J, Yacoub E, et al. A multi-modal parcellation of human cerebral cortex. Nature 2016;536:171–8.

[16] Behrens TEJ, Johansen-Berg H, Woolrich MW, Smith SM, Wheeler-Kingshott C a. M, Boulby PA, et al. Non-invasive mapping of connections between human thalamus and cortex using diffusion imaging. Nat Neurosci 2003;6:750–7. https://doi.org/10.1038/nn1075.

[17] Tziortzi AC, Haber SN, Searle GE, Tsoumpas C, Long CJ, Shotbolt P, et al. Connectivity-Based Functional Analysis of Dopamine Release in the Striatum Using Diffusion-Weighted MRI and Positron Emission Tomography. Cereb Cortex 2014;24:1165–77. https://doi.org/10.1093/cercor/bhs397.

[18] Diedrichsen J, Balsters JH, Flavell J, Cussans E, Ramnani N. A probabilistic MR atlas of the human cerebellum. NeuroImage 2009;46:39–46. https://doi.org/10.1016/j.neuroimage.2009.01.045.

[19] Keuken MC, Bazin P-L, Backhouse K, Beekhuizen S, Himmer L, Kandola A, et al. Effects of aging on T₁, T₂*, and QSM MRI values in the subcortex. Brain Struct Funct 2017;222:2487–505. https://doi.org/10.1007/s00429-016-1352-4.

[20] Guell X, Schmahmann J. Cerebellar functional anatomy: a didactic summary based on human fMRI evidence. Springer; 2020.

[21] Rubinov M, Sporns O. Complex network measures of brain connectivity: Uses and interpretations. NeuroImage 2010;52:1059–69. https://doi.org/10.1016/j.neuroimage.2009.10.003.

[22] Cox RW. AFNI: software for analysis and visualization of functional magnetic resonance neuroimages. Computers and Biomedical Research 1996;29:162–73.

[23] Kannurpatti SS, Biswal BB. Detection and scaling of task-induced fMRI-BOLD response using resting state fluctuations. Neuroimage 2008;40:1567–74. https://doi.org/10.1016/j.neuroimage.2007.09.040.

[24] Benjamini Y, Hochberg Y. Controlling the False Discovery Rate: A Practical and Powerful Approach to Multiple Testing. Journal of the Royal Statistical Society Series B (Methodological) 1995;57:289–300.

[25] Winkler AM, Ridgway GR, Webster MA, Smith SM, Nichols TE. Permutation inference for the general linear model. NeuroImage 2014;92:381–97. https://doi.org/10.1016/j.neuroimage.2014.01.060.
