# Supplementary material for: Sweet spot for resting-state functional MRI effect of deep brain stimulation in dystonia lies in the lower pallidal area
Source: Neuroimage Clin. 2025 Feb 5;45:103750. doi: 10.1016/j.nicl.2025.103750 (PMC11889665; doi:10.1016/j.nicl.2025.103750)

**Supplementary figure 1: Mask of sensorimotor regions**. Cortical areas presented as CIFTI grayordinate surface maps (left side of the picture). Subcortical structures shown in 4 slices z = 4, -10, -24, -38 in MNI coordinate system (right side of the picture). Combined parcellation based on HCP-derived cortical parcellation (180 parcels per hemisphere in total, 18 per hemisphere selected as sensorimotor function related) and subcortical area sub-segmentation based on several atlases (78 sub-segments in total, 40 selected as sensorimotor function related) – see Methods for more information.

**Supplementary figure 2: DBS electrode placement in eligible dystonia subjects for upper (red) and lower (blue) internal globus pallidus (GPi) area stimulation positions.** 3D point clouds of active stimulation contacts of individual subjects overlayed over 3D reconstruction of the right GPi (depicted in green) and ultra-high resolution ex vivo 7-Tesla MRI brain scan (Edlow et al. 2019). A) viewed in the latero-medial direction (from the right side), B) viewed in the postero-anterior direction (from behind). Stimulation contacts from the left hemisphere were non-linearly flipped to the right hemisphere; right hemisphere contacts depicted as are. 5 contacts in unsuitable positions where functional MRI signal was flipped from the contralateral hemisphere (see Methods for more information) not depicted


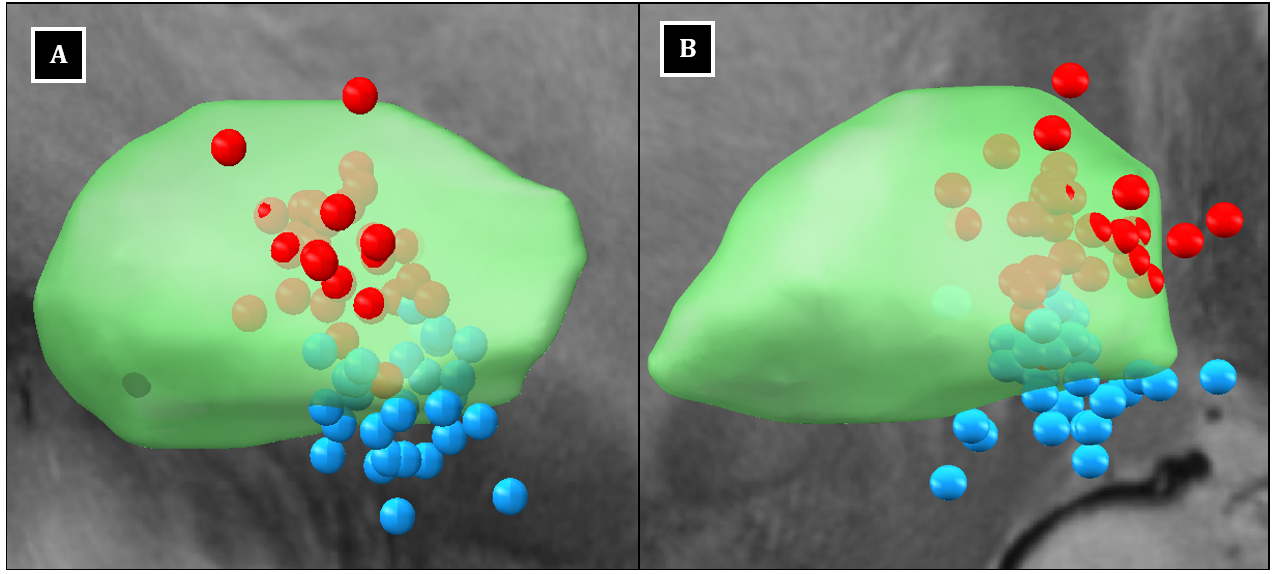


**Supplementary figure 3:** **Comparison of dystonia patients in the DBS OFF state** **and healthy controls for eigenvector centrality and RSFA**. Alpha of 0.05, false discovery rate corrected. Red-yellow scale marks showing the dystonia patients > healthy controls contrast; blue scale labels the reverse contrast; values correspond to the T statistic. Subcortical structures shown in 4 slices z = 4, -10, -24, -38 (MNI coordinate system). Laterality convention where the right side of the figure corresponds to the right side of the brain is used. See Supplementary table 2 for further anatomical and statistical information on significant regions. For the full information on individual parcels utilised as nodes, see (Glasser et al. 2016). Abbreviations: RSFA – resting state physiological fluctuation amplitude.


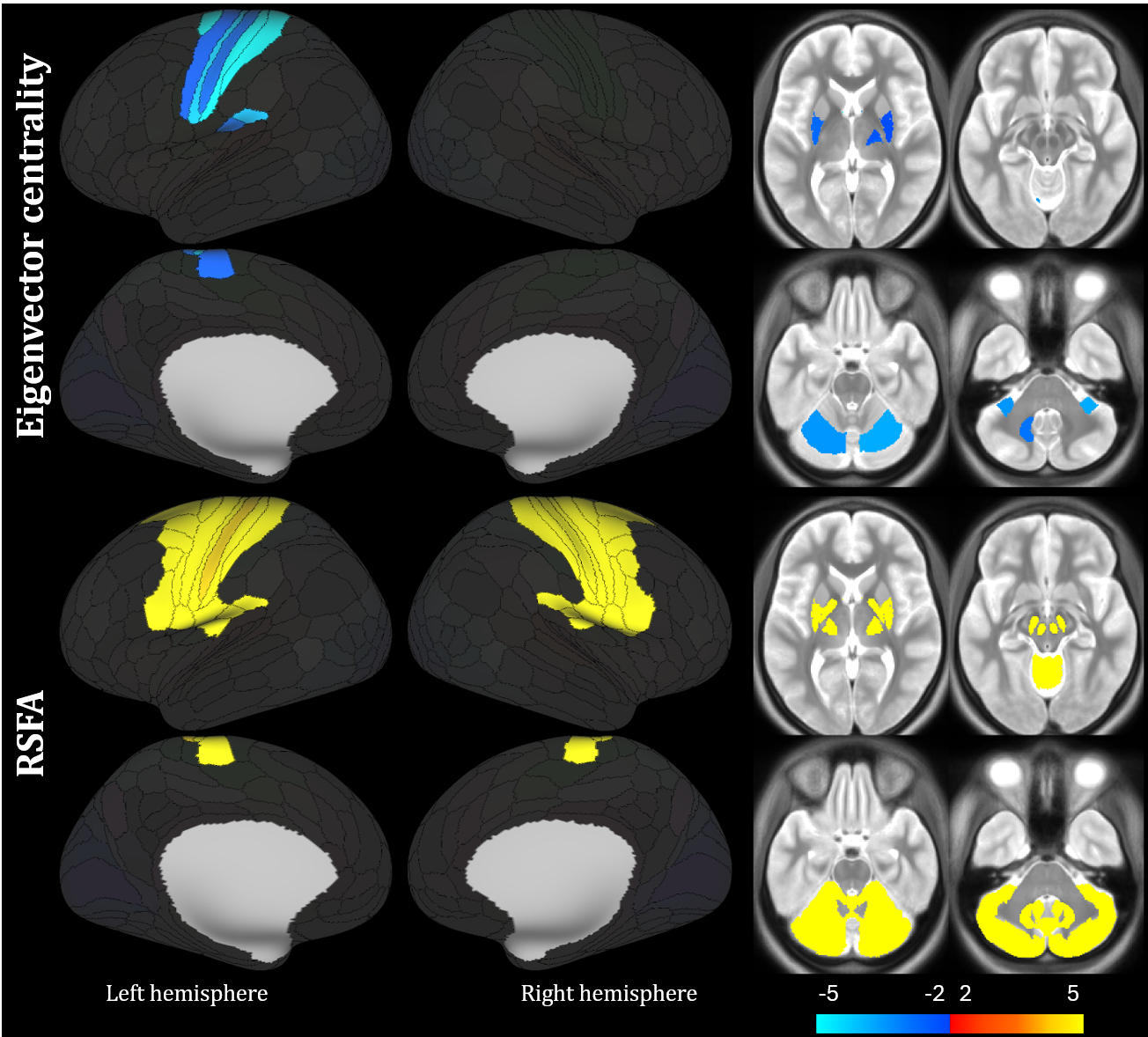


**Supplementary figure 4: Main results for the interaction analysis of the effect of the stimulation in the lower and upper GPi part vs DBS OFF state for RSFA and eigenvector centrality in a subset of 12 dystonia patients without hemisphere mirroring** due to unsuitable position of one of the stimulation contacts. Alpha of 0.05, **uncorrected**. Red-yellow scale marks showing the (lower GPi part vs DBS OFF) > (upper GPi part vs DBS OFF) contrast; blue scale labels the reverse contrast; values correspond to the T statistic. Subcortical structures shown in 4 slices z = 4, -10, -24, -38 (MNI coordinate system). Laterality convention where the right side of the figure corresponds to the right side of the brain is used. See Supplementary table XX for further anatomical and statistical information on significant regions. For the full information on individual parcels utilised as nodes, see (Glasser et al. 2016). Abbreviations: RSFA – resting state physiological fluctuation amplitude.


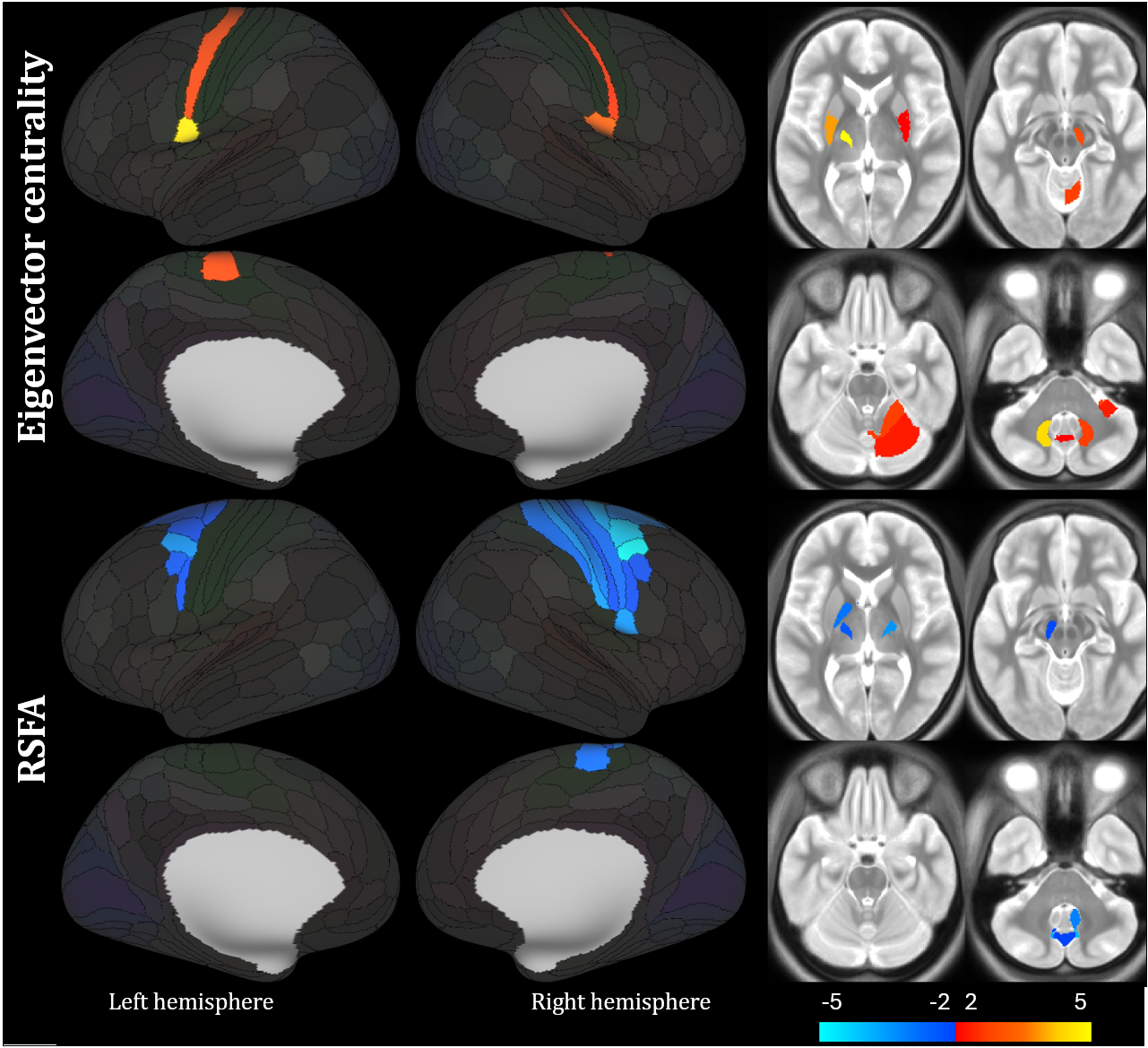


**Supplementary figure 5:** **Correlation of clinical score change in** **dystonia patients with eigenvector centrality, DBS ON in lower GPi part.** Eigenvector centrality for DBS ON in upper GPi part and RSFA not presented, since they failed to yield any statistically significant results. Alpha of 0.05, false discovery rate corrected. Red-yellow scale marks showing the positive correlation; blue scale labels the reverse contrast; values correspond to Pearson’s correlation coefficient. Subcortical structures shown in 4 slices z = 4, -10, -24, -38 (MNI coordinate system). Laterality convention where the right side of the figure corresponds to the right side of the brain is used. See Supplementary table 2 for further anatomical and statistical information on significant regions. For the full information on individual parcels utilised as nodes, see (Glasser et al. 2016).


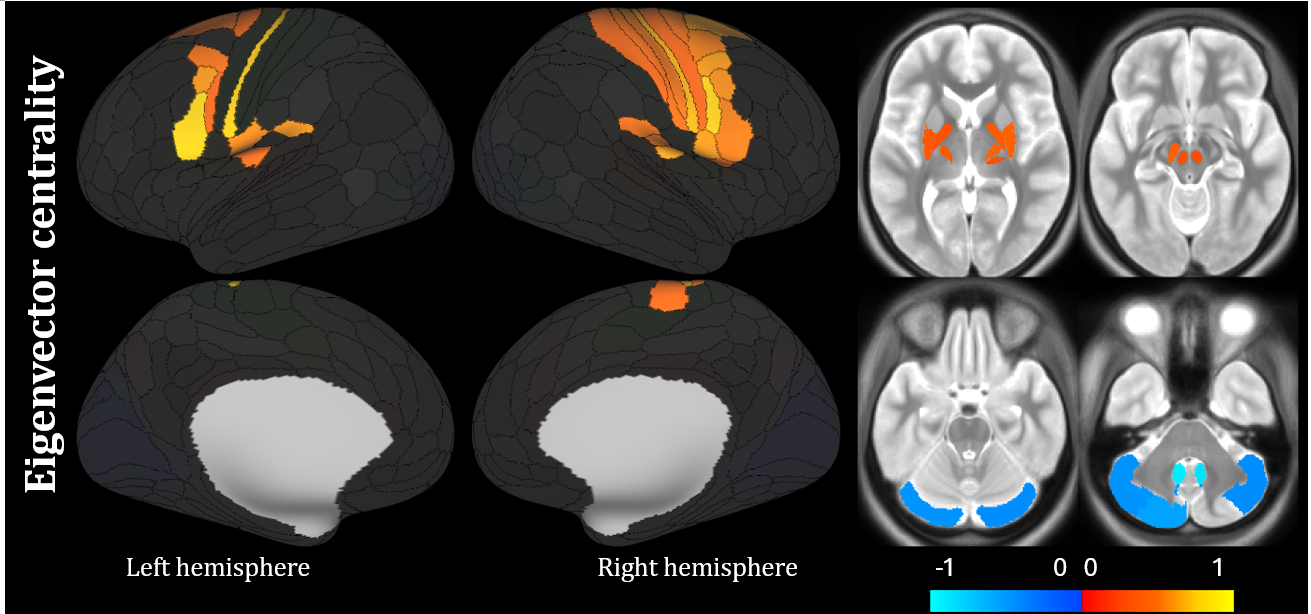

Supplement: Supplementary Data 2 [file mmc2.docx]
